# Internet-Delivered Dialectical Behavioral Therapy Skills Training for Chronic Pain: Protocol for a Randomized Controlled Trial

**DOI:** 10.2196/41890

**Published:** 2023-06-07

**Authors:** Nell Norman-Nott, Negin Hesam-Shariati, Chelsey R Wilks, Jessica Schroeder, Jina Suh, Nancy E Briggs, James H McAuley, Yann Quidé, Sylvia M Gustin

**Affiliations:** 1 NeuroRecovery Research Hub, School of Psychology University of New South Wales Sydney Australia; 2 Centre for Pain IMPACT Neuroscience Research Australia Sydney Australia; 3 NeuroRecovery Research Hub School of Psychology University of New South Wales Sydney Australia; 4 Centre for Pain IMPACT Neuroscience Research Australia Randwick Australia; 5 Department of Psychological Science University of Missouri-St Louis St. Louis, MO United States; 6 School of Computer Science and Engineering University of Washington Washington, WA United States; 7 Microsoft Research Redmond, WA United States; 8 Stats Central Mark Wainwright Analytical Centre University of New South Wales Sydney Australia; 9 School of Health Sciences University of New South Wales Sydney Australia

**Keywords:** internet-delivered, dialectical behavioral therapy, chronic pain, emotion dysregulation, emotion-centric intervention, mobile phone

## Abstract

**Background:**

Emotion dysregulation is key to the development and maintenance of chronic pain, feeding into a cycle of worsening pain and disability. Dialectical behavioral therapy (DBT), an evidence-based treatment for complex transdiagnostic conditions presenting with high emotion dysregulation, may be beneficial to manage and mitigate the emotional and sensory aspects of chronic pain. Increasingly, DBT skills training as a key component of standard DBT is being delivered as a stand-alone intervention without concurrent therapy to help develop skills for effective emotion regulation. A previous repeated-measure single-case trial investigating a novel technologically driven DBT skills training, internet-delivered DBT skills training for chronic pain (iDBT-Pain), revealed promising findings to improve both emotion dysregulation and pain intensity.

**Objective:**

This randomized controlled trial aims to examine the efficacy of iDBT-Pain in comparison with treatment as usual to reduce emotion dysregulation (primary outcome) for individuals with chronic pain after 9 weeks and at the 21-week follow-up. The secondary outcomes include pain intensity, pain interference, anxiety symptoms, depressive symptoms, perceived stress, posttraumatic stress, harm avoidance, social cognition, sleep quality, life satisfaction, and well-being. The trial also examines the acceptability of the iDBT-Pain intervention for future development and testing.

**Methods:**

A total of 48 people with chronic pain will be randomly assigned to 1 of 2 conditions: treatment and treatment as usual. Participants in the treatment condition will receive iDBT-Pain, consisting of 6 live web-based group sessions led by a DBT skills trainer and supervised by a registered psychologist and the iDBT-Pain app. Participants in the treatment-as-usual condition will not receive iDBT-Pain but will still access their usual medication and health interventions. We predict that iDBT-Pain will improve the primary outcome of emotion dysregulation and the secondary outcomes of pain intensity, pain interference, anxiety symptoms, depressive symptoms, perceived stress, harm avoidance, social cognition, sleep quality, life satisfaction, and well-being. A linear mixed model with random effects of individuals will be conducted to investigate the differences between the baseline, 9-week (primary end point), and 21-week (follow-up) assessments as a function of experimental condition.

**Results:**

Recruitment started in February 2023, and the clinical trial started in March 2023. Data collection for the final assessment is planned to be completed by July 2024.

**Conclusions:**

If our hypothesis is confirmed, our findings will contribute to the evidence for the efficacy and acceptability of a viable intervention that may be used by health care professionals for people with chronic pain. The results will add to the chronic pain literature to inform about the potential benefits of DBT skills training for chronic pain and will contribute evidence about technologically driven interventions.

**Trial Registration:**

Australian New Zealand Clinical Trials Registry ACTRN12622000113752; https://www.anzctr.org.au/Trial/Registration/TrialReview.aspx?id=383208&isReview=true

**International Registered Report Identifier (IRRID):**

PRR1-10.2196/41890

## Introduction

### Background and Rationale

Chronic pain, defined as any pain condition that persists continuously or intermittently beyond 3 months [1], is a substantial and costly burden on individuals and society [2,3]. Regardless of the condition (eg, arthritis, musculoskeletal pain, back pain, and fibromyalgia), chronic pain is a multidimensional construct incorporating both unpleasant sensory (eg, pain intensity) and psychological (eg, emotional) components [1]. Evidence corroborates the detrimental effect of chronic pain on emotions [4-7], demonstrating a cyclical association between negative emotional states, such as fear, anger, worry, and low mood, and worsening pain intensity and disability [8-10]. Beyond negative emotional states, co-occurring anxiety and depressive disorders present in up to 80% of people with chronic pain [11-14]. Other related issues include reduced sleep quality (up to 70%) and lower quality of life and well-being [15].

Increasingly, emotion dysregulation is regarded as a mechanism that underpins negative emotional states in people with chronic pain [16-18]. Emotion dysregulation is defined as an extreme sensitivity to emotional events and a reduced capacity to identify and moderate emotions [19]. The modal model of emotions helps make sense of the association between chronic pain and emotion dysregulation. According to this model, when a stimulus is experienced, it gives rise to an emotion that is then cognitively appraised before determining physiological arousal and a behavioral response [10,20]. For people with chronic pain, this process may be disrupted because emotion regulation capabilities are fatigued by persistent negative emotions that are likely a result of the debilitating and distressing symptoms of chronic pain and the experience of missing out because of episodes of escalating pain (eg, on career, social, and educational opportunities) [8-10]. Over time, emotion dysregulation appears to lead to a greater focus on pain, reports of heightened levels of pain intensity, and increasing negative emotions [21].

The development of emotion dysregulation may be further explained by previous experiences and neurobiological changes in people with chronic pain. The persistent presence of pain has been related to neurotransmitter dysregulation—specifically, a reduction in key neurochemicals (γ-aminobutyric acid and glutamate) in the medial prefrontal cortex—which is thought to affect social cognition, including the integration of emotions for decision-making, culminating in emotion dysregulation [22]. In addition, a recent mini review connects hyperactivity in emotion-regulating brain pathways with trauma, posttraumatic stress disorder (PTSD), and the presence and severity of chronic pain [23]. Trauma has been extensively reported as a risk factor for the development of chronic pain [21]. Emotion dysregulation may also be an antecedent to chronic pain, whereby certain personality traits manifest in emotion dysregulation, thereby increasing the risk of developing chronic pain [24,25]. Notably, chronic pain is a condition that is unpredictable in how it transpires over time, but regardless, psychological factors, specifically emotion dysregulation, appear to be a key factor in its development and maintenance [21].

Although a definitive explanation of how and why people with chronic pain present with emotion dysregulation remains elusive, the role of effective emotion regulation in reducing pain intensity and emotional distress is increasingly evident [26,27]. Successful emotion regulation includes emotional awareness, understanding and accepting emotions, and an ability to modify emotions or emotional expression consistent with social norms [19]. Existing psychological interventions for chronic pain, such as cognitive behavioral therapy, mindfulness-based stress reduction, and acceptance and commitment therapy, include few strategies for effective emotion regulation and have shown at best minimal improvement in mood and pain severity [28-30]. Including specific strategies to develop effective emotion regulation skills may mean that psychological interventions for chronic pain are more beneficial [16].

Owing to this promising evidence, psychological interventions for chronic pain that focus on improving emotion regulation are increasingly being investigated. Examples include interventions that incorporate emotion regulation training into cognitive behavioral therapy [31] and emotion awareness and expression therapy [32] and the integration of emotion regulation methods from dialectical behavioral therapy (DBT) [33]. A recent systematic review of randomized controlled trials (RCTs) summarized that emotion-regulation focused interventions, defined as studies that target emotion regulation as a mechanism of chronic pain, have a substantial clinical benefit in reducing pain; however, findings about psychological outcomes (eg, emotion dysregulation, depression, and anxiety) are uncertain owing to a lack of data [34]. Several pilot studies have begun to explore the effect of DBT on psychological outcomes, finding that DBT may improve anxiety and depression (in a study of 6 patients with chronic gastrointestinal pain [35]); depressive symptoms and emotion dysregulation (in a case study with a patient with chronic pain [36]); and negative emotion, fear, depression, and anxiety (seen in 5 out of 6 study participants with chronic lower back pain [37]). However, as these are all small pilot trials, further evidence is needed to understand the effects of psychological factors related to chronic pain in addition to pain intensity. Understanding how these types of interventions affect emotion dysregulation may be particularly important considering its potential role in the development and maintenance of chronic pain (as discussed previously).

Building on previous evidence of DBT for chronic pain, our group used one mode of DBT, DBT skills training, to develop a 6-session internet-delivered intervention focused on improving emotion dysregulation in people with chronic pain. Standard DBT is an evidence-based treatment for clients with multiple diagnoses presenting with high emotion dysregulation [38]. Standard DBT encompasses a comprehensive treatment plan using 4 modes delivered over 12 months: group skills training, individual therapy, phone coaching, and a therapist consultation team. However, increasingly, DBT skills training as a key mode of standard DBT is delivered as a stand-alone intervention without the 3 other modes [39]. An advantage of stand-alone DBT skills training is that the resources and time to deliver it are substantially reduced, whereas a real-world study found that improvements in emotion dysregulation are comparable with those of standard DBT (ie, all modes) [40]. In addition, DBT skills training does not necessarily require a psychologist; it can be delivered by DBT trainers, who can be anyone well trained on the principles of the training and skills (eg, teachers, professional trainers, volunteers, therapists, physiotherapists, and nurse practitioners) [39,41].

The DBT skills training developed for this trial is the internet-delivered DBT skills training for chronic pain (iDBT-Pain), a guided intervention designed to enhance emotion regulation through learning key skills from DBT in mindfulness, emotion regulation, and distress tolerance. These modules are first taught by a DBT skills trainer and a supervising psychologist in weekly sessions and then reinforced and practiced by accessing an app. As an intervention that is optimized for mobile use (eg, on a smartphone or tablet), iDBT-Pain is functional for anyone with a mobile device and an internet connection. To our knowledge, iDBT-Pain is the first guided internet-delivered DBT intervention for people with chronic pain even though technologically supported interventions have been encouraged since a Cochrane report in 2014 [42]. Importantly, internet-delivered psychological interventions increase accessibility for remote communities, where it can be difficult to access mental health services, and enable access for people with restricted mobility because of chronic pain [42].

A previous trial using a repeated single-case experimental design (SCED) explored the efficacy of iDBT-Pain, showing promising findings to improve both emotion dysregulation and pain intensity with effect sizes of −0.88 and −0.81, respectively [43]. However, although the findings of this previous trial are encouraging, further evidence is necessary to compare iDBT-Pain to usual modes of treatment in a larger sample to generalize the findings more broadly. This protocol details the methods to be used in an RCT to investigate the efficacy of iDBT-Pain. This trial will test a group-based version of iDBT-Pain that builds on the individualized treatment previously investigated in the SCED trial. Group treatment has advantages over individualized treatment, including enabling individuals to interact with others like themselves, resulting in validation that can in itself be therapeutic [39].

Overall, this trial builds on the literature on interventions that focus on improving emotion regulation as a key mechanism related to chronic pain. This trial will add evidence to the current literature on the efficacy and acceptability of a potentially viable emotionally focused intervention that may be administered by health care professionals for people with chronic pain.

### Choice of Comparators

To investigate the efficacy of iDBT-Pain, we chose treatment as usual (TAU) as a comparator, with the open-ended definition of TAU as any treatment received for chronic pain that an individual accesses from their usual health care provider. The rationale for this comparator is that we consider it necessary to compare the efficacy of iDBT-Pain with those treatment options that would be available in the absence of the intervention. In addition, TAU as a comparator assists with the feasibility of the study as there is no need to recruit expert clinicians on a specific comparator therapy method. Therefore, the chosen trial design enables a feasible comparison of the efficacy of iDBT-Pain with the general practice in chronic pain treatment and enables the investigation of whether iDBT-Pain can come close to the effects of the multifaceted, individually adjusted chronic pain treatment currently delivered by health care services in the community.

### Objectives

#### Primary Objective

Our primary objective is to evaluate the efficacy of iDBT-Pain in reducing emotion dysregulation (primary outcome) in individuals with chronic pain. The 9-week iDBT-Pain intervention is hypothesized to improve emotion dysregulation compared with TAU.

#### Secondary Objectives

First, we will investigate the efficacy of iDBT-Pain in improving the secondary outcomes of pain intensity, pain interference, anxiety symptoms, depressive symptoms, perceived stress, posttraumatic stress, social cognition, sleep quality, life satisfaction, and well-being. It is hypothesized that the group administered the 9-week iDBT-Pain intervention will improve in the secondary outcomes compared with the TAU control group.

Second, we will investigate the efficacy of the iDBT-Pain intervention at the 21-week follow-up on the primary and secondary outcomes. It is hypothesized that the iDBT-Pain intervention group will significantly improve in both the primary and secondary outcomes compared with the TAU control group.

Finally, we will investigate the acceptability of the iDBT-Pain intervention at the 9- and 21-week follow-ups. It is anticipated that these findings will help determine participants’ affective and cognitive responses to the intervention as well as indicate the most useful aspects of the intervention for future development and testing.

### Trial Design

In a 2-arm randomized controlled superiority trial, participants with chronic pain will be allocated at a ratio of 1:1 to either receive iDBT-Pain (in addition to TAU) or continue with TAU only. Assessments will take place at baseline and the 9-week (primary end point) and 21-week follow-ups. The efficacy of iDBT-Pain will be determined by comparing treatment with TAU for the primary and secondary outcomes. The acceptability of iDBT-Pain will be investigated using a mixed methods approach whereby quantitative and qualitative data (semistructured interviews) will be collected, separately analyzed, and then collectively interpreted [44]. Reporting of this protocol is in accordance with the SPIRIT (Standard Protocol Items: Recommendations for Interventional Trials) guidelines [45] (Multimedia Appendix 1).

## Methods

### Study Setting

This study will be conducted by researchers in Australia at the University of New South Wales (UNSW). The iDBT-Pain intervention will be administered by researchers using videoconferencing and an app, meaning that it will be accessed entirely on the web by participants in their everyday life. Assessments, including participant screening for eligibility, will be conducted remotely via telephone and links to web-based questionnaires.

### Eligibility Criteria

Participants will be eligible for this trial if they fulfill the inclusion and exclusion criteria outlined in Table 1.

**Table 1:** Inclusion and exclusion criteria.

	Inclusion criteria	Exclusion criteria
Age	Adults aged >18 years	Individuals aged <18 years
Chronic pain	Average pain rating of ≥3 for the past 7 days (Numeric Rating Scale)	Average pain rating of <3 for the past 7 days (Numeric Rating Scale)
Technology	Access and capability to use the internet to participate in the iDBT-Pain^a^ sessions and access to a smartphone or tablet device capable of running the iDBT-Pain app	No access or capability to use the internet or no access to an internet-enabled device (eg, smartphone or tablet)
Commitment	Ability to commit to the expectations of the trial (eg, weekly sessions lasting 90 minutes and daily practice)	Inability to commit to the expectations of the trial
Psychiatric disorders	No diagnosed psychotic or personality disorders	Diagnosed psychotic (eg, schizophrenia) or personality (eg, borderline personality disorder or bipolar disorder) disorders; uncontrolled mental health disorder
Dementia or Alzheimer disease	No presence of dementia or Alzheimer disease	Diagnosis of dementia or Alzheimer disease
Language	Reasonable level of written and spoken English	Poor level of written and spoken English
Country of residence	Located in Australia	Located outside Australia

^a^iDBT-Pain: internet-delivered dialectical behavioral therapy skills training for chronic pain.

### Informed Consent Process

Informed consent will be obtained after potential participants have been screened as eligible (see the *Recruitment* section) and stated their interest in participating in the trial and before they complete the baseline questionnaires. A trained research assistant or a member of the research team will contact the potential participant by email to arrange a time for a call to discuss informed consent. Before the call, the potential participant will be sent a link to the participant information statement and consent form to ensure that they have the full details about the trial and consent process ahead of the call. During the call, the opportunity to ask questions about the trial and consent will be emphasized, and the procedure and opportunity to withdraw from the trial will be fully explained. To ensure understanding and that the questions have been addressed adequately, a teach-back method of informed consent will be used whereby the researcher will ask the potential participant to restate information back in their own words. Consent will be obtained only from individuals with the capacity to make an informed decision regarding their participation. Potential participants who wish to consent to participate will be sent a link to sign the consent form in a secure web-based application (Qualtrics [Qualtrics International Inc]). This form will then be countersigned by the principal investigator, and a copy will be emailed to the participant for their records.

### Additional Consent Provisions

On the consent form, participants can optionally consent to (1) their anonymized data being used in future research, (2) being contacted about future research studies for which they might be suitable, (3) being contacted about potential participation in media events, (4) allowing video recording of the iDBT-Pain Zoom (Zoom Video Communications) sessions, (5) being entered into a prize draw if they complete the study, and (6) using the iDBT-Pain app after the study. For more details, copies of the participant information statement and consent form are available upon request from the corresponding author. This trial does not involve collecting biological specimens.

### Interventions

#### Treatment Group

**Figure 1:**
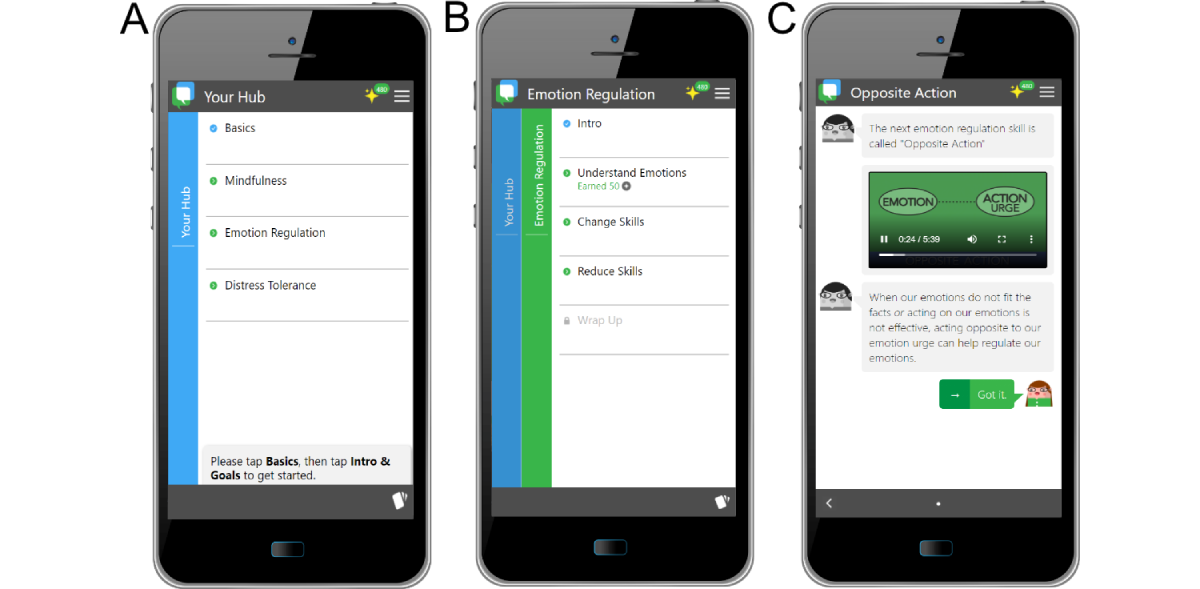
Screenshots of the key sections within the internet-delivered dialectical behavioral therapy skills training for chronic pain (iDBT-Pain) app. (A) iDBT-Pain app home page; (B) the 3 emotion regulation modules; and (C) learning the skill of opposite action.

Participants in the treatment group will receive the iDBT-Pain intervention (Table 2), consisting of two key elements: (1) the iDBT-Pain sessions, involving six 60- to 90-minute iDBT-Pain sessions delivered via Zoom to groups of 8 to 12 participants, and (2) the iDBT-Pain app, accessed by participants on their own smart devices (eg, smartphone) to watch videos and complete tasks (Figure 1). In addition to the iDBT-Pain sessions and app, there will be an introductory session to explain the trial and a concluding session. To develop iDBT-Pain [46], evidence-based protocols for DBT skills training according to the DBT Skills Training Manual [39] were used. Participants in the treatment group may continue to receive their usual care (eg, medication and physiotherapy) while in the trial; any such treatment will be monitored (see the *Medication and Health Intervention Measures* section).

The intervention will be delivered by a DBT skills trainer who is a psychology graduate, PhD candidate, and qualified in DBT skills from the Linehan Institute [41]. An Australian Health Practitioner Regulation Agency–registered psychologist will supervise the DBT trainer. Both the trainer and supervisor are familiar with the iDBT-Pain intervention, having delivered it in the previous trial [43], and will adhere to the intervention manual. To ensure treatment fidelity and manual adherence, with the participants’ permission, Zoom sessions will be recorded.

**Table 2:** Internet-delivered dialectical behavioral therapy skills training for chronic pain (iDBT-Pain) intervention protocol.

Week	iDBT-Pain session content (via Zoom [Zoom Video Communications])	iDBT-Pain app content
1	Introductory session: meet the skills trainers and the group; intervention overview; and association between chronic pain, the brain, and emotions	N/A^a^
2	Session 1—mindfulness 1: rationale for mindfulness for chronic pain and skills of *wise mind* and mindfulness *whats*	Introduction to mindfulness; *Wise mind*; 3 mindfulness *whats* skills (observe, describe, and participate); and watching the video about the association between chronic pain, emotions, and the brain
3	Session 2—mindfulness 2: mindfulness *how* skills to practice mindfulness with skillful effectiveness	Mindfulness *how* skills (nonjudgmentally, one mindfully and effectively)
4	Session 3—emotion regulation 1: how to identify and label emotions as a function for emotion regulation	Introduction to emotion regulation and understanding and naming emotions
5	Session 4—emotion regulation 2: skills to reduce the frequency and quantity of unwanted emotions	Handling unwanted emotions
6	Session 5—emotion regulation 3: skills to build future resilience against intense emotion	Building mastery and coping ahead
7	Session 6—distress tolerance: skills that help weather crises and intense negative emotions	Distress tolerance skills: TIP^b^ and distract
8	Concluding session: recap the skills learned and the association between chronic pain and difficulties with emotions	Train daily in all skills for mindfulness, emotion regulation, and distress tolerance
9	No session	Train daily in all skills for mindfulness, emotion regulation, and distress tolerance

^a^N/A: not applicable.

^b^TIP: temperature of face, intense exercise, and paced breathing.

#### Control Group

Participants in the control group will receive TAU, meaning that they may receive treatment options offered by their usual health care provider (eg, general practitioner). Treatment received outside the trial will be monitored and described [47].

### Criteria for Discontinuing or Modifying the Allocated Interventions

Dropout from the study will be recorded, including the reasons. Participants will be free to withdraw at any point. Withdrawn participants will not be replaced. If a participant chooses to withdraw, the research team will only contact them to ascertain reasons for not wishing to take part or continue and ask if they would still be willing to take part in the assessments to enable the intention-to-treat (ITT) analysis.

In the case of an adverse event, an evaluation form will be completed by the research team for the principal investigator in accordance with the UNSW Safety Monitoring Register Template. The form will include a description of the event; a classification of seriousness; an assessment of the potential relationship to the intervention; and, if considered serious, an assessment of the need for change in the consent or the study activities, a summary of known previous health issues, and the event outcome. This process will inform whether the adverse event warrants a trial or treatment modification or discontinuation by a participant.

### Strategies to Improve Adherence

To improve adherence to the intervention, at the outset of the trial, all participants in the treatment group will be emailed a calendar invite to book the time for the weekly Zoom sessions in their diary and provide them with the Zoom link. Participants will then be contacted (eg, by email) with a reminder the day before the weekly Zoom session. Participants will be asked to attend all Zoom sessions live unless there are circumstances where they are unable to do so (eg, they have an urgent appointment to attend). On occasions in which a participant cannot attend the live session, after the Zoom session has finished, they will be contacted by the DBT skills trainer, emailed a link to a secure password-protected web page that will contain a recording of the Zoom session, and asked to watch the video before the next weekly Zoom session. To ensure adherence to using the app daily, all participants will be sent detailed instructions to set up the app on their smartphone at the outset of the trial and will be reminded in each of the weekly Zoom sessions to continue to practice in the app for homework. Participants allocated to TAU will receive access to the app after completing the questionnaires in the study. Participants in the TAU group will also be reminded of the importance of their contribution to the research to try to avoid resentment.

### Relevant Concomitant Care Permitted or Prohibited During the Trial

Participants who are currently taking medication (eg, pain-relieving analgesics) and receiving therapeutic interventions (eg, physiotherapy) will be eligible for the trial. Medication and intervention use will be documented (see the *Secondary Outcome Measures* section) at the 3 assessment time points (baseline, 9-week follow-up, and 21-week follow-up). All patients will be encouraged to continue their usual treatments that they access for their pain.

### Ancillary Provisions and Posttrial Care

The clinical trial insurance held by the trial sponsor (UNSW) provides indemnity against personal injury claims from trial participants arising from their involvement in a UNSW-sponsored human clinical trial.

### Outcome Measures

#### Overview

In addition to clinical and demographic data assessed at baseline (eg, age, sex, education, marital status, pain condition, and pain location), outcomes, measures, and their assessment time points are presented in Table 3. All measures used for the primary and secondary outcomes have demonstrated validity and reliability. Measures to monitor participants’ ongoing medication and health interventions accessed during the trial are also listed. All measures, including demographic data, will be self-reported by the participants on the web. Links to the questionnaires will be emailed to participants at 3 time points: baseline, 9-week follow-up, and 21 week follow-up.

**Table 3:** Measures and time points of administration to participants.

Domain	Measure	Time point		
		Baseline	9-week follow-up	21-week follow-up
Emotion dysregulation^a^	Difficulties in Emotion Regulation Scale–18	✓	✓	✓
Pain intensity^b^	Numeric Rating Scale	✓	✓	✓
Pain interference^b^	PROMIS^c^ Pain Interference instrument	✓	✓	✓
Anxiety^b^	State-Trait Anxiety Inventory	✓	✓	✓
Depression^b^	Beck Depression Inventory	✓	✓	✓
Perceived stress^b^	NIH^d^ Toolbox Perceived Stress Scale	✓	✓	✓
Posttraumatic stress^b^	PTSD^e^ Checklist–Civilian Version	✓	✓	✓
Social cognition^b^	The Awareness of Social Inference Test	✓	✓	✓
Sleep quality^b^	Medical Outcomes Study Sleep Scale	✓	✓	✓
Harm avoidance^b^	Temperament and Character Inventory	✓	✓	✓
Quality of life^b^	Satisfaction with Life Scale	✓	✓	✓
Well-being^b^	COMPAS-W^f^ scale	✓	✓	✓
Medication and health interventions	Health Utilization Questionnaire	✓	✓	✓
Medication and health interventions	Medication and health interventions	✓	✓	✓
Acceptability^b,g^	DBT^h^ Ways of Coping Checklist (DBT skills)	✓	✓	✓
Acceptability	Usefulness, Satisfaction, and Ease of Use Questionnaire		✓	✓
Acceptability	Patient Global Impression of Change		✓	✓
Acceptability	Semistructured interviews		✓	
Acceptability	Treatment adherence		✓	✓

^a^Primary outcome.

^b^Secondary outcome.

^c^PROMIS: Patient-Reported Outcomes Measurement Information System.

^d^NIH: National Institutes of Health.

^e^PTSD: posttraumatic stress disorder.

^f^COMPAS-W: Composure, Own-worth, Mastery, Positivity, Achievement, Satisfaction with Life–Well-being.

^g^Using the listed quantitative and qualitative measures, seven domains of acceptability will be assessed according to the theoretical framework of acceptability [48]: (1) affective attitude, (2) burden, (3) ethicality, (4) intervention coherence, (5) opportunity costs, (6) perceived effectiveness, and (7) self-efficacy.

^h^DBT: dialectical behavioral therapy.

#### Primary Outcome Measure

The primary outcome of emotion dysregulation will be measured using the Difficulties in Emotion Regulation Scale [49], an 18-item Likert-type scale ranging from 1 (*almost never*) to 5 (*almost always*) with some reverse-coded items. Total scores will be used for this study, with higher scores indicating a higher level of emotion dysregulation.

#### Secondary Outcome Measures

##### Numeric Rating Scale

Pain intensity will be measured using the Numeric Rating Scale [50], a rating scale ranging from 0 to 10, with 0 representing one extreme (*no pain*) and 10 representing the other extreme (*pain as bad as it could be*) over the last 7 days.

##### Patient-Reported Outcomes Measurement Information System Pain Interference Instrument

Pain interference will be measured with the Patient-Reported Outcomes Measurement Information System Pain Interference instrument [51], an 8-item Likert-type scale ranging from 1 (*not at all*) to 5 (*very much*), to assess how much pain has interfered with life in the last 7 days. Total raw scores will be converted to a standardized score, with higher scores representing higher pain interference.

##### State-Trait Anxiety Inventory

State anxiety will be measured using the state anxiety subscale of the State-Trait Anxiety Inventory [52], a 20-item Likert-type scale ranging from 1 (*not at all*) to 4 (“very much so”), to measure current anxiety. Higher scores denote higher anxiety.

##### Beck Depression Inventory

Depressive symptoms will be measured using the Beck Depression Inventory [53], a scale where participants select the best-fitting statement from 4 options ranging from 0 to 3 to measure depressive symptoms experienced in the last 7 days. Higher scores reflect greater depressive symptoms.

##### National Institutes of Health Toolbox Perceived Stress Scale

Perceived stress will be measured using the National Institutes of Health Toolbox Perceived Stress Scale [54], a 10-item Likert-type scale with scores ranging from 1 (*never*) to 5 (*very often*), to assess the levels of stress experienced in the past month. Total raw scores will be converted to a standardized score, with higher scores representing higher stress.

##### PTSD Checklist–Civilian Version

Posttraumatic stress symptoms will be measured using the PTSD Checklist–Civilian Version [55], a 17-item Likert-type scale with scores ranging from 1 (*not at all*) to 5 (*extremely*), to assess how much participants have been bothered by problems regarding past traumatic events over the last month. Total scores will be used, with higher scores indicating a higher level of posttraumatic stress symptoms (re-experiencing, avoidance, and hyperarousal).

##### The Awareness of Social Inference Test–Short

Social cognition will be measured using a web-based version of the Awareness of Social Inference Test–Short [56]. The Awareness of Social Inference Test–Short uses audiovisual vignettes separated into three parts: (1) The Emotion Evaluation Test, in which participants identify emotions (happy, neutral, sad, anxious, angry, and disgusted); (2) the Social Inference–Minimal; and (3) the Social Inference–Enriched. For parts 2 and 3, participants are asked to answer *Yes*, *No* or *Don’t Know* to 4 probe questions about thoughts, intentions, and feelings. The 3 parts are scored, and there is an overall score (sum of the 3 parts), with higher scores indicating higher social cognition.

##### Medical Outcomes Study Sleep Scale

Sleep quality will be measured using the Sleep Problems Index II from the Medical Outcomes Study Sleep Scale [57], a 9-item Likert-type scale ranging from 1 (*all of the time*) to 6 (*none of the time*), to measure sleep quality over the last 4 weeks. Higher scores indicate greater sleep problems over the last 4 weeks.

##### Temperament and Character Inventory–Harm Avoidance Scale

The personality trait of harm avoidance will be measured using the Temperament and Character Inventory–Harm Avoidance Scale [58], a 35-item scale that uses alternate-response questions (true or false) to measure how individuals usually feel. Total scores are converted to percentile scores (84%-100%=very high; 67%-83.3%=high; 34%-66.7%=average; 17%-33%=low; 0%-16.7%=very low).

##### Satisfaction With Life Scale

Overall life satisfaction will be measured using the Satisfaction with Life Scale [59], a 5-item Likert-type scale ranging from 1 (*strongly disagree*) to 7 (*strongly agree*). Scores are summed to obtain a total, with higher scores indicating higher life satisfaction.

##### The Composure, Own-Worth, Mastery, Positivity, Achievement, Satisfaction With Life–Well-being Scale

Well-being will be measured using the Composure, Own-worth, Mastery, Positivity, Achievement, Satisfaction with Life–Well-being Scale [60], a 26-item Likert-type scale ranging from 1 (“strongly disagree”) to 5 (*strongly agree*) with 5 items reverse-coded (items 5, 7, 14, 21, and 26). Participants’ scores are summed and compared with standardized cutoffs: languishing (<88), moderate (89-108), and flourishing (>109).

### Acceptability Measures

A mixed methods (qualitative and quantitative) approach will be used to measure acceptability in accordance with the theoretical framework for acceptability (TFA) [48]. The TFA encompasses seven domains to measure cognitive and emotional responses to a health care intervention: (1) affective attitude, (2) burden, (3) ethicality, (4) intervention coherence, (5) opportunity costs, (6) perceived effectiveness, and (7) self-efficacy [48].

#### Semistructured Interview

A semistructured interview will be administered via video conference at 9 weeks to participants one-on-one in the treatment group. The question topics in the semistructured interview will be guided by the TFA [48]. Specifically, questions will provide insights into affective attitude (eg, “what are your thoughts about the program?”), burden (eg, “Has the effort required to participate in the intervention outweighed the burden of participating?”), ethicality (eg, “How likely would you be to recommend the program?”), intervention coherence (eg, “What’s been your experience of using the web app?”), self-efficacy (eg, “How confident are you in your abilities to use the intervention?”), opportunity costs (eg, Have you had to give up anything that is of benefit to you to do the intervention?), and perceived effectiveness (eg, “How does this approach compare to other treatments? Has it had more effect/less?”).

#### Usefulness, Satisfaction, and Ease of Use Questionnaire

The Usefulness, Satisfaction, and Ease of Use Questionnaire [61] is a 30-item Likert-type scale ranging from 1 (*strongly disagree*) to 7 (*strongly agree*) whereby average scores are calculated for 4 dimensions of usability (usefulness, ease of use, ease of learning, and satisfaction). A higher mean reflects a greater presence of the dimension, with a suggested threshold score of 5 denoting a positive rating [62]. The TFA constructs of burden, self-efficacy, intervention coherence, and ethicality will be assessed using the Usefulness, Satisfaction, and Ease of Use Questionnaire subscales of usefulness, ease of use, ease of learning, and satisfaction, respectively.

#### DBT Ways of Coping Checklist–DBT Skills Subscale

The DBT skills subscale of the DBT Ways of Coping Checklist [63], which evaluates DBT skills use, will be used to help evaluate intervention coherence. An average score across the subscale items will be calculated, with higher average scores indicating greater intervention compliance.

#### Patient Global Impression of Change Scale

Perceived effectiveness will be measured using the Patient Global Impression of Change Scale [64], a 7-point scale depicting overall improvement related to activity limitations, symptoms, emotions, and overall quality of life, and a scale of 0 (*much better*) to 10 (*much worse*) points to identify the degree of change since the start of treatment.

#### Treatment Adherence

The burden of the intervention will be evaluated according to session attendance (eg, iDBT-Pain sessions attended) and time spent using the app, recorded by the DBT skills trainer and on the app server. A higher attendance and greater amount of time spent on the app will indicate a lower burden.

### Medication and Health Intervention Measures

Although participants will be asked to refrain from making changes to their current medication (eg, pain analgesics) and health care treatment (eg, physiotherapy) during the trial, it is realistic that the treatment that participants access from their regular health care providers may change. To capture information about medication and health care changes, participants will be asked to complete two measures: (1) the Chronic Pain Medication and Health Intervention Questionnaire and (2) the Healthcare Utilization Questionnaire.

### Participant Timeline

Interested participants will undergo a structured screening process that includes a web-based screening survey and, for those eligible after their survey responses, a phone call with a study investigator. Phone screening will be conducted as soon as possible after the web-based screening. After obtaining informed consent, baseline questionnaires will be administered within a window of 3 weeks before randomization. Participants will be randomly allocated and will learn about group assignment at least 1 week before the first Zoom session. After the 9-week intervention delivery period, participants will be assessed again at 9 and 21 weeks after randomization. Participants will be asked to complete the assessment within 1 week and will be prompted by phone, SMS text message, and email a maximum of 3 times for up to 4 weeks if they are not responsive. See Table 4 for a schematic showing the schedule of enrollment, interventions, and assessments.

**Table 4:** Participant timeline (SPIRIT [Standard Protocol Items: Recommendations for Interventional Trials]) showing the schedule of enrollment, interventions, and assessments.

				Study period								
				Enrollment		Baseline and allocation		Postallocation				
Time point				−12 to −1 week		−2 to −1 week		0-9 weeks (intervention)		9-week follow-up		21-week follow-up
**Enrollment**												
	Eligibility screening (online and phone)		✓									
	Informed consent		✓									
	Allocation				✓							
**Interventions**												
	iDBT-Pain^a^						✓					
	Treatment as usual						✓					
**Assessments**												
	**Primary outcome measure**											
		Difficulties in Emotion Regulation Scale-18			✓				✓		✓	
	**Secondary outcome measures**											
		Numeric Rating Scale			✓				✓		✓	
		PROMIS^b^ Pain Interference Instrument			✓				✓		✓	
		State-Trait Anxiety Inventory			✓				✓		✓	
		Beck Depression Inventory			✓				✓		✓	
		NIH^c^ Toolbox Perceived Stress			✓				✓		✓	
		PTSD^d^ Checklist–Civilian Version			✓				✓		✓	
		The Awareness of Social Inference Test			✓				✓		✓	
		Medical Outcomes Study Sleep Scale			✓				✓		✓	
		Temperament & Character Inventory			✓				✓		✓	
		Satisfaction with Life Scale			✓				✓		✓	
		COMPAS-W^e^ Scale of Wellbeing			✓				✓		✓	
		Health Utilization Questionnaire			✓				✓		✓	
		Medication & Health Interventions			✓				✓		✓	
	**Acceptability measures**											
		DBT^f^ Ways of Coping Checklist			✓				✓		✓	
		Usefulness, Satisfaction & Ease of Use							✓		✓	
		Patient Global Impression of Change							✓		✓	
		Semistructured interview							✓			
		Treatment adherence					✓		✓		✓	

^a^iDBT-Pain: internet-delivered DBT skills training for chronic pain.

^b^PROMIS: Patient-Reported Outcomes Measurement Information System.

^c^NIH: National Institutes of Health.

^d^PTSD: posttraumatic stress disorder.

^e^COMPAS-W: Composure, Own-worth, Mastery, Positivity, Achievement, Satisfaction with Life–Well-being.

^f^DBT: dialectical behavioral therapy.

### Sample Size

The sample size for this trial was calculated using the SAS software (version 9.4; SAS Institute) of the SAS system (SAS and all other SAS Institute Inc product or service names are registered trademarks or trademarks of SAS Institute Inc in the United States and other countries) and is based on the large effect size (pain intensity; effect size=0.81) obtained from our previous trial [46]. The power analysis is based on the effect size of the secondary outcome from the previous trial rather than the primary outcome effect size of 0.88 for emotion dysregulation to obtain a more cautious sample size estimate given that the previous trial was conducted with a small population using a single-case design. To detect a large mean difference (η^2^=0.14) between the treatment groups in this trial, a total of 40 people (20 in each group) will be required. This assumes 80% power, a Cronbach α of .05, and a within-subject correlation of 0.5. Attrition rates for studies investigating cognitive behavioral treatments for chronic pain vary between 0% and 30%, with a mean attrition of 13% (SD 10%); thus, we have set expectations for attrition at 20%, resulting in a total sample size of 48 [65]. We expect this estimate to be transferable to the web-based delivery of psychological interventions as research suggests that trials using remote delivery generally show comparable or even lower rates of attrition [66]. Rates of attrition will be monitored by the research team at regular milestones during the trial (eg, at the point where 50% of the participants have reached their scheduled 21-week follow-up time), and we will consider taking steps to increase sample size if this is required.

### Recruitment

Individuals with chronic pain who had previously contacted our group (n=300) and requested information about participating in studies on chronic pain will receive by email the study details, including the participant information statement, and a link to the study web page. Recruitment will also use ethically approved web-based community advertising on Facebook. There is growing evidence that Facebook is a useful recruitment tool for research, particularly to access hard-to-reach populations [67] such as the chronic pain population. The Facebook advertisement will include a link to the study web page.

From the study web page, interested participants can click through to a web-based screening survey that will ask questions about age and demographics, chronic pain condition, existing and previous psychological and neurological diagnoses, internet accessibility, fluency in reading and speaking English, contact details, and location. Potential participants who are positive on their web-based screening will be contacted to arrange a phone call with a study investigator. The purpose of the phone call is to ask further questions to determine eligibility. Questions will ascertain chronic pain diagnosis, injury, current medications, therapy, and interventions being received, along with plans for any upcoming medication or treatment changes or surgery. An important part of the screening phone call is to determine whether there is a previous or current diagnosis of any psychological or neurological disorder that may exclude the participant from the study. The researcher will ask the potential participant if there is any current or previous diagnosis of schizophrenia, borderline personality disorder, or bipolar disorder and whether there is a current diagnosis of a psychological disorder that is uncontrolled (eg, depression that is not being treated with psychotherapy or antidepressants). Those who do not meet the eligibility criteria will be informed of this sensitively and asked if they wish to remain on our secure database to be contacted about future trials.

**Figure 2:**
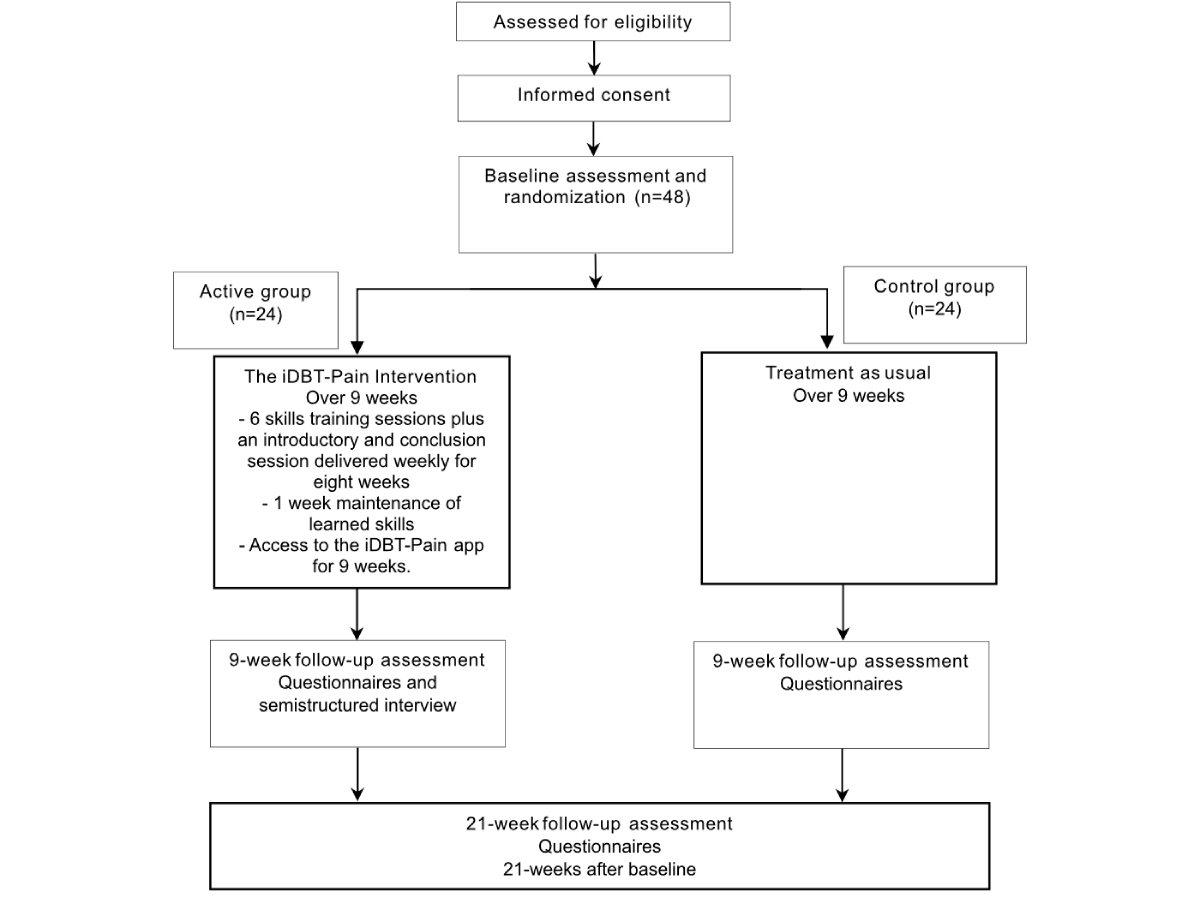
Flowchart of the study design. iDBT-Pain: internet-delivered dialectical behavioral therapy skills training for chronic pain.

Participants that are eligible will be emailed after the phone call to complete the informed consent process, whereby they will have the opportunity to discuss the Participant Information Statement and consent process with a researcher before electronically signing the consent form. Eligible, fully informed, and consenting participants will then be entered into the study for baseline assessment and randomization (Figure 2).

### Allocation

#### Sequence Generation

Following informed consent, participants will be randomly allocated to groups (treatment or TAU) at a ratio of 1:1. A block randomization schedule will be used with a block size of 2 generated by a statistician blinded to study recruitment, treatment, and data collection. Opaque sealed envelopes will ensure concealment from the DBT trainer and psychologist administering the intervention until the interventions are assigned. An independent research assistant will prepare the envelopes. Once the participant has consented to the trial and completed the baseline assessment, the independent research assistant will open the envelope to assign the participant. Owing to the nature of this intervention, the participants and DBT skills trainer delivering the intervention will not be blinded to group allocation. The skills trainer will be made aware of the group allocation by the research assistant and will then advise the participants of their group after completion of the baseline assessment. The statistician conducting the primary data analysis will be blinded to group allocation.

#### Concealment Mechanism

Opaque sealed envelopes will ensure concealment until interventions are assigned.

#### Implementation

A statistician blinded to study recruitment, treatment, and data collection will generate the allocation sequence. Participants will be enrolled in the study by the researchers, who will be blinded until after intervention assignment. An independent research assistant will assign participants to the interventions.

### Blinding

Owing to the nature of this intervention, the participants and those delivering the intervention (DBT skills trainer and psychologist) will not be blinded to group allocation after the baseline assessment. However, the baseline assessment is conducted before randomization, meaning that there is no risk of disclosure of treatment allocation to the assessor or participant at the time. All assessments will be distributed remotely via automated email with a web link to complete the assessment on the web, thereby ruling out any potential effects of assessors on assessments of outcomes at the 9- and 21-week follow-ups. The statistician analyzing the outcome data will remain blind to treatment allocation throughout the analysis.

### Data Collection and Management

#### Plans for Assessment and Collection of Outcomes

Data will be collected sequentially using web-based questionnaires administered through the Qualtrics survey platform at 3 time points: baseline and 9- and 21-week follow-ups. The measures for the primary and secondary outcomes are validated and commonly used instruments (see the *Outcome Measures* section). Web-based data collection is an appropriate way of collecting data for our study as questionnaires can be answered and delivered remotely, consistent with the web-based nature of the trial. Participants will self-report data about their pain, emotions, and psychological states and traits.

#### Plans to Promote Participant Retention and Complete Follow-up

To aid retention, participants in both groups who complete the trial may enter a prize draw to receive 1 of 2 Aus $200 (US $133.72) gift cards. To further aid retention, participants (treatment and TAU) who complete the study (ie, following the 21-week follow-up assessment) will be asked if they would like to retain or obtain access to the iDBT-Pain app. In addition, the use of web-based assessments will reduce the burden on participants to promote retention. Dropout from the study will be recorded, including the reasons. Participants will be free to withdraw at any point. Withdrawn participants will not be replaced. Upon conclusion of the study (ie, following the 21-week follow-up assessment), all participants (treatment and TAU) will be asked if they would like to retain or obtain access to the iDBT-Pain app.

### Data Management

The research team will treat all participant information ethically and with confidentiality in accordance with the UNSW data governance policy. Data will be anonymized and stored on a secure data platform at UNSW protected via username and password. Personal and identifiable information will be stored separately from the anonymized trial data, and data will be anonymized wherever possible. For analysis, the qualitative and statistical data will be downloaded onto password-protected computers accessible by the trial investigators. Data from the web-based questionnaires will be quality checked by the research team before analysis.

### Confidentiality

Information collected as part of the trial will be kept strictly confidential within the research team. Participants’ names and contact details will be stored in password-protected files on secure servers and separately to linked anonymized data. To ensure skills trainer adherence and competency, the sessions will be recorded with the consent of all participants appearing in the recording. With the consent of the participants, recordings will be made available to participants who need to catch up on missed content on a password-protected web link to view the video on the web. Participants will have the option to keep their camera off during the session. Access to these recordings is restricted to those with the link and password.

### Access to Data

All investigators will be able to access the full trial data set. To ensure confidentiality, data dispersed to the project team members and the statistician conducting the analysis will not contain any identifying participant information.

### Data Analysis Methods

#### Statistical Methods

##### Overview

For all primary and secondary outcomes that are normally distributed and measured over time, a linear mixed model with random effects of individuals will be specified. The fixed effects of time, treatment group, and their interaction will be included. Treatment differences at 9 and 21 weeks and change from baseline to 9 and 21 weeks within each group will be estimated. If the distributional assumption of normality is not tenable, an appropriate transformation of the outcome will be conducted. Alternatively, a generalized linear mixed model with appropriate distribution will be used.

The data will be analyzed on an ITT basis. Unadjusted and adjusted analyses will be conducted. The adjusted analyses will include age at baseline and sex. *P* values of <.05 will be considered significant. Effect sizes will be expressed in terms of pooled baseline SD units, as described by Feingold [68].

##### Qualitative Methods

Qualitative data from the semistructured interviews will be transcribed, coded, and then summarized according to the TFA framework [48]. Specifically, under the headings of the TFA constructs (affective attitude, burden, perceived effectiveness, ethicality, intervention coherence, opportunity costs, and self-efficacy), a thematic analysis will be used to code responses according to key emerging themes [69]. Quantitative data from measures that assess domains of acceptability along with treatment adherence data will be descriptively summarized according to the individual measures. A collective summary of both quantitative and qualitative data will be reported to provide a comprehensive understanding of the acceptability of the iDBT-Pain intervention.

##### Methods for Additional Analyses

There is no a priori plan to conduct subgroup analyses.

##### Methods in Analysis to Handle Protocol Nonadherence and Any Statistical Methods to Handle Missing Data

Although linear mixed models do allow for unbiased estimates of the treatment effect with missing data assuming data that are missing at random [70], we will also use multiple imputation to estimate the treatment effect in the primary outcome as a sensitivity analysis. Supposing that the missing-at-random assumption is tenable, we will use multiple imputation using chained equations, include variables of baseline pain intensity and psychological factors in the imputation model, and then estimate the treatment effect in the imputed data.

As a sensitivity analysis, a modified ITT analysis will be performed. As detailed in the *Allocation* section, participants are randomized into a treatment group, but they will be included in the final data set only if they have completed the 6 iDBT-Pain skills training sessions delivered via Zoom in weeks 2 through 7 either by attending the session or by watching a video of the session.

### Data Monitoring

An independent data monitoring committee has not been arranged as the intervention occurs over a short period, the protocol will not be modified irrespective of data collected during the intervention, and there are minimal risks to participants. No interim analyses are planned.

### Trial Management Group

The Trial Management Group (TMG) will oversee the day-to-day conduct of the trial and will be responsible for reviewing the safety information to identify any serious emerging safety concerns. Any major amendments required to the study or changes to the protocol will first be agreed upon by the TMG and then communicated to participants as soon as possible by one of the researchers. The TMG will be responsible for seeking the advice of the ethics committee and the trial sponsor. The TMG is a board of elected individuals comprising the principal investigator, researchers, DBT skills trainers, trial managers, and research assistants.

### Adverse Event Reporting and Harms

DBT skills training is an evidence-based protocol for emotion dysregulation [39,71] and a standardized approach with no known side effects [35,36]. Thus, although we will measure safety, we do not anticipate any intervention- or study-related adverse events or reactions and consider the potential risks to be mild and of minimal likelihood to occur. Potential risks include experiencing temporary and transient increases in distress during the skills training, discomfort in terms of fatigue from attending up to 9 hours of Zoom sessions over the course of the intervention, and upset or discomfort when answering the questionnaires. To identify risk issues, participants will have the ability to communicate with the research staff throughout the study via email and phone, with concerns then raised to the TMG. Where necessary, participants will be provided with details of support services or advised to contact their regular health care professional (eg, general practitioner). To mitigate fatigue during the Zoom sessions, the sessions will be spaced evenly throughout the intervention period, 1 per week over 8 weeks, with sufficient time between sessions and breaks as necessary. Participants will also be asked to have a glass or bottle of water during the Zoom sessions to stay hydrated. If participants find the questionnaires distressing, they will be advised that they can skip to the next question or stop immediately.

Safety will be measured according to adverse events (untoward medical occurrence) or reactions (unintended response to treatment) related to iDBT-Pain, with data collected about the incidents by the research team in trial-related Safety Monitoring and Adverse Events forms.

### Frequency and Plans for Auditing Trial Conduct

The TMG will be responsible for periodic auditing to evaluate trial conduct and compliance with the protocol, good clinical practice, standard operating procedures, and UNSW sponsor and ethics requirements.

### Protocol Amendments

In the instance that amendments to the protocol are needed, the TMG will seek sponsor approval, after which trial registrations and the published protocol will be amended accordingly. A member of the TMG will communicate any resulting changes in the protocol to the participants.

### Dissemination Plans

To enable effective dissemination, the results will be considered for submission to appropriately selected peer-reviewed journals, summarized in a press release for the media, and included in presentations at conferences. This will ensure the dissemination of this research and data to other researchers and interested parties. Anonymized data sets and associated material may be available upon reasonable request from the corresponding author. Participants will be sent a summary of the research findings if they consent to this.

### Ethics Approval and Consent to Participate

Ethics approval was obtained from the University of New South Wales Human Ethics Committee (HC220078). Written informed consent to participate will be obtained from all participants.

## Results

Recruitment started in February 2023, and the clinical trial started in March 2023. Data collection for the final assessment is planned to be completed by July 2024.

## Discussion

### Potential Clinical Implications

Emotion dysregulation is key to the development and maintenance of chronic pain, leading to a cycle of worsening pain intensity, disability, and emotional difficulties [16-18]. Evidence is arising that DBT may be a viable intervention to target emotion dysregulation in people with chronic pain, helping reverse this cycle of pain and emotional distress and leading to improved physical and psychological function [35-37]. Specifically, iDBT-Pain, a guided internet-delivered approach encompassing live web-based sessions and access to an app, has been found in a previous SCED trial to reduce symptoms of emotion dysregulation and pain intensity [46].

In this protocol, we described an RCT to test iDBT-Pain compared with a control group. We plan to investigate the efficacy of iDBT-Pain in improving the primary outcome of emotion dysregulation and the secondary outcomes of pain intensity, pain interference, anxiety symptoms, depressive symptoms, perceived stress, posttraumatic stress, harm avoidance, social cognition, sleep quality, life satisfaction, and well-being. The RCT will test the acceptability of a group-based version of iDBT-Pain that builds on the individualized treatment previously investigated in the SCED trial.

To our knowledge, iDBT-Pain is the first guided internet-delivered DBT intervention for people with chronic pain. As an internet-delivered approach, iDBT-Pain offers an accessible option for those with restricted mobility and remote communities where there are often limited psychological services for people with chronic pain. If our hypotheses are confirmed, our findings will contribute to the evidence for the efficacy and acceptability of a viable intervention that may be used by health care professionals for people with chronic pain. Similarly, the results will add to the chronic pain literature to inform about the potential benefits of DBT and technologically driven interventions. More broadly, the findings will contribute to the evidence on whether interventions that target emotion dysregulation in people with chronic pain can lead to improved emotions and less pain.

### Limitations

The limitations of this study include the potential for participants to access other interventions and pain analgesics while in the trial. However, we will ask participants to disclose their pain medication and treatments and advise us of any changes in their dosages that occur during the trial period. Thus, the results can be considered in the context of any confounding factors related to treatment outside the trial. A further limitation is that therapist blinding is not possible because of the nature of the intervention. In addition, the sample size may limit the ability to generalize the findings to a larger population and detect more subtle effects.

## Appendix

Multimedia Appendix 1 SPIRIT (Standard Protocol Items: Recommendations for Interventional Trials) checklist.

## Abbreviations

DBT: dialectical behavioral therapy
iDBT-Pain: internet-delivered dialectical behavioral therapy skills training for chronic pain
ITT: intention-to-treat
PTSD: posttraumatic stress disorder
RCT: randomized controlled trial
SCED: single-case experimental design
SPIRIT: Standard Protocol Items: Recommendations for Interventional Trials
TAU: treatment as usual
TFA: theoretical framework for acceptability
TMG: Trial Management Group
UNSW: University of New South Wales

## Data Availability

Anonymized data sets and associated material will be available upon reasonable request from the corresponding author.
